# Supplementary material for: Arabic translation and cultural adaptation of sedentary behavior, dietary habits, and preclinical mobility limitation questionnaires: A cognitive interview study
Source: PLoS One. 2023 Jun 12;18(6):e0286375. doi: 10.1371/journal.pone.0286375 (PMC10259774; doi:10.1371/journal.pone.0286375)
Supplement: S1 Appendix — (DOCX) [file pone.0286375.s001.docx]

**Sedentary behavior Questionnaire (SBQ)**

| On **a typical WEEKDAY** | None | 15  min or less | 30  min | 1  hrs | 2  hrs | 3  hrs | 4  hrs | 5  hrs | 6  hrs or more |
| --- | --- | --- | --- | --- | --- | --- | --- | --- | --- |
| Watching television (including videos on VCR/DVD) | **□** | **□** | **□** | **□** | **□** | **□** | **□** | **□** | ___hrs |
| Playing computer or video games | **□** | **□** | **□** | **□** | **□** | **□** | **□** | **□** | ___hrs |
| Sitting listening to music on the radio, tapes, or CDs | **□** | **□** | **□** | **□** | **□** | **□** | **□** | **□** | ___hrs |
| Sitting and talking on the phone | **□** | **□** | **□** | **□** | **□** | **□** | **□** | **□** | ___hrs |
| Doing paperwork or computer work (office work, emails, paying bills, etc) | **□** | **□** | **□** | **□** | **□** | **□** | **□** | **□** | ___hrs |
| Sitting reading a book or magazine | **□** | **□** | **□** | **□** | **□** | **□** | **□** | **□** | ___hrs |
| Playing a musical instrument | **□** | **□** | **□** | **□** | **□** | **□** | **□** | **□** | ___hrs |
| Doing artwork or crafts | **□** | **□** | **□** | **□** | **□** | **□** | **□** | **□** | ___hrs |
| Sitting and driving/riding in a car, bus, or train | **□** | **□** | **□** | **□** | **□** | **□** | **□** | **□** | ___hrs |

**English (Weekday)**

On **a typical** **WEEKDAY**, how much time do you spend (from when you wake up until you go to bed) doing the following

**English (Weekend)**

On **a typical WEEKEND**, how much time do you spend (from when you wake up until you go to bed) doing the following

| **On a typical WEEKEND** | None | 15  min or less | 30  min | 1  hrs | 2  hrs | 3  hrs | 4  hrs | 5  hrs | 6  hrs or more |
| --- | --- | --- | --- | --- | --- | --- | --- | --- | --- |
| Watching television (including videos on VCR/DVD) | □ | □ | □ | □ | □ | □ | □ | □ | ___hrs |
| Playing computer or video games | □ | □ | □ | □ | □ | □ | □ | □ | ___hrs |
| Sitting listening to music on the radio, tapes, or CDs | □ | □ | □ | □ | □ | □ | □ | □ | ___hrs |
| Sitting and talking on the phone | □ | □ | □ | □ | □ | □ | □ | □ | ___hrs |
| Doing paperwork or computer work (office work, emails, paying bills, etc) | □ | □ | □ | □ | □ | □ | □ | □ | ___hrs |
| Sitting reading a book or magazine | □ | □ | □ | □ | □ | □ | □ | □ | ___hrs |
| Playing a musical instrument | □ | □ | □ | □ | □ | □ | □ | □ | ___hrs |
| Doing artwork or crafts | □ | □ | □ | □ | □ | □ | □ | □ | ___hrs |
| Sitting and driving/riding in a car, bus, or train | □ | □ | □ | □ | □ | □ | □ | □ | ___hrs |

# **Diet**

Questions adapted from Survey of Health, Aging and Retirement in Europe (SHARE) study

## Diary Product

**In the past month, how often do you have a serving of dairy products such as a glass of milk, cheese in a sandwich, a cup of yogurt or a can of high protein supplement?**

- Every day
- 3-6 times a week
- Twice a week
- Once a week
- Less than once a week

## Legumes and Eggs

**In the past month, how often do you have a serving of legumes (200g), beans (3 tablespoons) or eggs (2 eggs/serving)?**

- Every day
- 3-6 times a week
- Twice a week
- Once a week
- Less than once a week

## Meat

**In the past month, how often do you eat meat, fish or poultry?**

- Every day
- 3-6 times a week
- Twice a week
- Once a week
- Less than once a week

## Fruits and Vegetable

**In the past month, how often do you consume a serving of fruits or vegetables?**

*A serving of fruit: 2 or more small fruit – for example, 2 plums, 2 satsumas, 2 kiwi fruit, 3 apricots, 6 lychees, 7 strawberries or 14 cherries.

*A serving of vegetable: 2 broccoli spears or 4 heaped tablespoons of cooked kale, spinach, spring greens or green beans.

- Every day
- 3-6 times a week
- Twice a week
- Once a week
- Less than once a week

## Mobility limitations

Questions on performance-based measures of mobility (1)

**Do you have difficulty in walking 2.0km?**

- Able to manage without difficulty
- Able to manage with some difficulty
- Able to manage with great deal of difficulty
- Able to manage only with help of another person
- Unable to manage even with help

**Do you have difficulty in walking 0.5km?**

- Able to manage without difficulty
- Able to manage with some difficulty
- Able to manage with great deal of difficulty
- Able to manage only with help of another person
- Unable to manage even with help

**Do you have difficulty in walking 1 flight of stairs?**

- Able to manage without difficulty
- Able to manage with some difficulty
- Able to manage with great deal of difficulty
- Able to manage only with help of another person
- Unable to manage even with help
