# Supplementary material for: Arabic translation and cultural adaptation of sedentary behavior, dietary habits, and preclinical mobility limitation questionnaires: A cognitive interview study
Source: PLoS One. 2023 Jun 12;18(6):e0286375. doi: 10.1371/journal.pone.0286375 (PMC10259774; doi:10.1371/journal.pone.0286375)
Supplement: Attachment — Submitted filename: Reviewer report.docx [file pone.0286375.s002.docx]

**Title: Sedentary Behavior, Dietary Habits, and Preclinical Mobility Limitation Questionnaires: Translation and Cross-Cultural Adaptation to Arabic Population”**

**Reviewer's report:**

Thanks for allowing me to review your interesting manuscript titled " Sedentary Behavior, Dietary Habits, and Preclinical Mobility Limitation Questionnaires: Translation and Cross-Cultural Adaptation to Arabic Population”

Dear Authors,

kindly find the following points that need for your careful review and work on your research

article quality improvement:

**Introduction:**

- ProPASS: This is in need for operational definition by the meaning and the reason for selected that area for data collection.

- Theoretical background regarding Sedentary Behavior, Dietary Habits, and Preclinical Mobility Limitation current situation in Arabic population to be expanded in the introduction section. It is recommended to clarify the theoretical background of your research variables based on the aim the research.

- **The research question: not found**

**Methods:**

- Complete research design is required to be clarified in details

- The sample calculation and the technique of sampling is required to be clarified.

- This word (researcher) should be replaced by the authors or researchers

-	pilot sample not clear.

- The tools reliability and validity tests need to be clarified.

- please mention the exact time of sampling when began and ended.

**Results:**

- Table (1) needed to revised because percentage with frequency not correct.

- The tables 2,3,4 can be collected in one table and reorganized and what about other questions in cognitive interview such as, are there any activities or examples that we omitted?

**Discussion:**

- The discussion section should align by the results section and add interpretation about the

findings and support that interpretation with the different international studies, but actually it

miss that and needs for improvement.

-Limitation of study should be in separated section.

- Implication of study should be write.
